# Stochastic resonance analysis of a coupled high-speed maglev vehicle-bridge coupled system under bounded noise

**DOI:** 10.1038/s41598-023-33202-2

**Published:** 2023-05-09

**Authors:** Yan-xia Li, Zhi-wu Yu, Lei Xu

**Affiliations:** 1 Central South University, Changsha, 410075 China; 2 National Engineering Center of High-Speed Railway Construction, Changsha, 410075 China

**Keywords:** Engineering, Mathematics and computing

## Abstract

Coupled oscillations typically occur in maglev vehicle-bridge coupled systems excited by bounded noise caused by guideway irregularities. The paper employed Hamilton equations to derive the corresponding canonical transformation equations and determined the critical stable regions for two kinds of resonances using the largest Lyapunov exponents. The results show that the critical stable region between the excitation amplitude and the resonant frequency ratio is a valley shape when the system has external resonance only. When considering both internal and external resonances, the critical stable region between the excitation amplitude and resonant frequency ratio presents a small saddle shape. Energy transfers from the first to the second oscillator under with both internal and extrinsic resonance. As the guideway irregularities’ coefficients increase, the maximum Lyapunov exponents of the two conditions change from negative to positive, which means that the system varies from a stable state to instability.

## Introduction

Electromagnetic suspension (EMS) system^1^ is becoming increasingly popular in urban transportation due to its high speed. Compared with wheel/rail trains which are typically propelled by motors and adhesive forces, maglev vehicles are suspended in the air through electromagnetic induction ^2–5^ and are more sensitive to internal excitation, external excitation, air gap floating, circuit fluctuations, and bridge vibrations. Therefore, suppression methods for resonances have encountered various challenges such as moving loads, dynamic deflection, heterogeneous frequencies, invalid controllers, faulty suspension magnets, bridge’s lightweight, and unmatched indices ^6^.

The test results of the Shanghai maglev line and the German EMSLAND line show that the dynamic loads’ factor of the two-span track beam is less than 1.2 and the local dynamic loads’ factor is less than 1.5 over the whole speed range. To prevent the vehicle-bridge coupled system’s resonance, the DIN (Deutsche Industrie Norm) standards stipulate that the product of the beam’s fundamental frequency and a single hole span need to be equal to 1.1 times the speed limit. It has been observed that the natural frequencies of the suspension system and the track are equal to 4.8 Hz and 17 Hz, respectively. However, because the track has a large oscillation amplitude^7^, the resonance needs to be calculated based on thorough analyses.

In this paper, a series of approaches for calculating resonance were presented including the average power for moving distribution loads’ method and the Lyapunov method. The average power technique was based on the transfer function and the stable condition described by Li Jinhui^8–10^, who provided the minimum model of the maglev vehicle-bridge interaction system, the necessary conditions for its stability, and three principles underlying the self-excited vibration. The resonant conditions under moving distribution loads were improved by Fryba, Yau J. D., Kwark, Xia H., and Yang Y. B.^11–15^, who gave the critical speeds at which the resonance may occur, thought the maximum acceleration responses of the beam to be dominated by the fundamental vibration modals, presented the numerical method technique concerning the dynamic behavior of bridges, explained the mechanisms of resonances and cancellation, and proposed the resonant formulas for calculating the span and frequency. The effect of fuzzy controller was discussed by Sun Yougang^16^, which can improve the dynamic performance of the system, make the maglev system obtain a large stable range, and restrain the vehicle–guideway interaction vibration effectively.

Noise is commonly considered undesirable. However, some special nonlinear systems have nonintuitive dynamic behaviors after noise is introduced. Indeed, in recent years, there have been several studies to demonstrate the phenomenon of stochastic resonance (SR). Examples were: (i) an experiment demonstrating stochastic resonance in a bistable electronic device: a tunnel diode ^17^. Stochastic resonance was detected using a simple experimental setup by investigating the time evolution of the voltage measured across the tunnel diode as a function of the input noise intensity. (ii) the stochastic resonant phenomena was studied experimentally and theoretically for a state-of-the-art metal-oxide memristive device based on yttria-stabilized zirconium dioxide and tantalum pent-oxide, which has exhibited bipolar filamentary resistive switching of the anionic type ^18^. The optimal noise intensity corresponding to the stochastic resonance phenomenon was interpreted using a stochastic memristor model by adding an external noise source to the control voltage. Furthermore, dynamical systems have been studied in the presence of relevant noise-induced phenomena with a constructive role in stability, such as, (i) the damping-enhanced stability of a Brownian particle starting from an unstable initial position and moving in a metastable system was explored ^19^; (ii) a memristor used for resistive switches behaved as multistable nonlinear systems between low-resistance and high-resistance states in a random telegraphic signal mode ^20^; (iii) an approach using a real stable polynomial combined with a Gauge transformation was presented and the bistability of polynomials corresponding to factors of the original multi-graph model resulted in real stable polynomials of each factor in various multi-graph models of the aforementioned contraction sequence ^21^; (iv) a nonstationary function within a memristive system was investigated to devise a simplified description of transient processes under different noise intensities, and the relaxation time was obtained, which depended nonmonotonically on the intensity of the fluctuations ^22^. Finally, in other scientific study fields, such as quantum phase transitions in complex biological and physical systems, the positive role of noise has also been demonstrated. For example: (i) the Gaussian non equilibrium steady states of the quantum characteristics of such critical phenomena have been reviewed ^23^. (ii) a quantum case has been detected in which the indeterminacy arising from the uncertainty principle reduced the accuracy of the parameter estimation in a way that cannot be neglected, even in the limit of infinite copies^24^. (iii) the phenomena of dissonance and consonance have considered, where two sensory neurons were driven by noise and subthreshold periodic signals, and their outputs plus noise were applied to a third neuron with noise added to them ^25^; (iv) the noise in the high resistive state was found to be featured by nearly the same probability density functions and spectrum as the inner noise of the experimental setup ^26^.

Compared with previous research frameworks of time-domain samples in Newtonian mechanics, a Hamiltonian system with narrow-band random excitation is more complex. Some theoretical bases have been proposed. Colored noise refers to a fixed centre frequency, white noise intensity, and a uniform distribution angle with a triangular relationship ^27, 28^, which examined the responses. This noise was utilized in a Wiener process by an equivalent to measured power spectra in methods put forward by Chen Zeshen, Jin Zhibin, and Jin Shi ^29–31^, who performed the theoretical modeling analysis in the time domain with covariance analysis method, generated guideway irregularities by combining the shape filter with the time delay system, and considered short-wavelength track irregularities. Some examples substantiated in bridge responses under wind loads have been obtained by Dimentberg M, Lin Y.K., and Jian Deng. ^32–34^, who obtained the subcritical responses to an external broadband random, considered turbulence stabilize even a single-degree of freedom structural motion, and provided insights on how to analyze and control parametric resonances under a bounded noise process in engineering applications. Since Lyon et al. first applied a stochastic averaging method^35^ proposed by R. L. Stratonovich^36^, it was subsequently applied by Zhu W. Q., Huang Z. L., Liu Zhonghua, and W.Y. Liu^37, 38^, who proposed a stochastic averaging method to predict approximately the response of quasi-integrable Hamiltonian systems excited by bounded noise, determined the threshold of bounded noise amplitude for the onset of chaos. They have applied to duffer oscillator analyses using the random mean principle and the limited differential technique. Although Bo Zhang^39^ investigated the random stability of a suspended wheelset system considering Gaussian white noise by the random average method. At present, there are few studies on resonance based on stochastic stability. Solving the resonant behaviour of the complex maglev vehicle-bridge coupled system is key to the further development of EMS.

The study presented in this paper aims to build a model to explore the critical conditions of stochastic resonance over the whole bridge span with the aerodynamic loads and guideway irregularities. Hamilton’s theory is applied to derive the differential equations and their dimensionless equations ^28^. The appropriate stable domains at different resonances based on stochastic averaging theory and canonical transformations are given. The stability probability according to the Fokker–Planck–Kolmogorov (FPK) equation utilized in this study. Moreover, a unique numerical method for assessing the effects of aerodynamic loads and the guideway excitation on the stochastic resonance of the maglev vehicle-bridge coupled system is also presented.

## The stochastic averaging method

To explain the theoretical basis of our model’s analysis, the derivation process of the stochastic averaging method is introduced below.

Consider a quasi-integrable Hamiltonian system under bounded noise excitation governed by the following equations of motion^37^:

1 $$\dot{{Q}_{i}}=\frac{\partial H}{\partial {P}_{i} } \,\,\dot{{P}_{i}}=-\frac{\partial H}{\partial {Q}_{i}}-\varepsilon {c}_{ij}\frac{\partial H}{\partial {P}_{i} }+\varepsilon {h}_{ij}{\xi }_{k}\left(t\right)  \,\, i,j=1,...,n;k=1,...,l$$

where ${Q}_{i}$ and ${P}_{i}$ are the generalized displacements and momentum, respectively; *H* = *H*(*Q*, *P*) is the Hamiltonian; $\varepsilon {c}_{ij}=\varepsilon {c}_{ij}(Q, P)$ are the coefficients of lightly linear or nonlinear damping; $\varepsilon {h}_{ij}=\varepsilon {h}_{ij}(Q,P)$ denotes the amplitudes of weak bounded noises; and ${\xi }_{k}(t)$ represents independent bounded noises of the form

2 $${\xi }_{k}\left(t\right)=cos\left[{\Omega }_{k}t+{\sigma }_{k}{B}_{k}\left(t\right)+{\Delta }_{k}\right]\,\,k=1,...,l$$

where ${\Omega }_{k}$ and ${\sigma }_{k}^{2}$ are constants representing the center frequencies and strengths of the frequency perturbations, respectively; ${B}_{k}(t)$ are independent units in the Wiener processes; and ${\Delta }_{k}$ are independent random phases that are uniformly distributed in [0, 2π]. ${\xi }_{k}(t)$are independent stationary random processes in a wide sense with spectral densities

3 $${S}_{k}(\omega )=\frac{{\sigma }_{k}^{2}}{4\pi }\frac{{\sigma }_{k}^{2}+{\omega }^{2}+{\sigma }_{k}^{4}/4}{({\omega }^{2}-{\Omega }_{k}^{2} -{\sigma }_{k}^{4}/4)+{\sigma }_{k}^{4}{\omega }^{2}}$$

and auto correlation functions

4 $${R}_{k}(\tau )=\frac{1}{2 }exp(-\frac{{\sigma }_{k}^{2}}{2}|\tau |)cos{\Omega }_{k}\tau$$

The bandwidths of the processes ${\xi }_{k}(t)$ depend mainly on the parameters ${\sigma }_{k}$. The processes are narrow-banded when ${\sigma }_{k}$ are small and wide-banded processes when ${\sigma }_{k}$ are large. It is assumed that ${\sigma }_{k}$ are small and thus the corresponding processes are narrow-band.

Suppose that the Hamiltonian system shown with Eq. (1) with $\varepsilon =0$ is integrable, i.e., there exists a set of canonical transformations

5 $${I}_{i}={I}_{i}(Q,P), {\theta }_{i}={\theta }_{i}(Q,P) \,\,i=1,...,n$$

through which new Hamiltonian equations are of  the following form:

6 $$\dot{{\theta }_{i}}=\frac{\partial H(I)}{\partial {I}_{i} }={\omega }_{i}(I)\,\,\dot{{I}_{i}}=-\frac{\partial H(I)}{\partial {\theta }_{i}}=0 \,\,i=1,...,n$$

where ${I}_{i}$ and ${\omega }_{i}$ are action variables and frequencies, respectively; ${\theta }_{i}$  are the angle variables conjugated to ${I}_{i}$; and $H(I)$ is the transformed Hamiltonian, which is independent of ${\theta }_{i}$. The Hamiltonian system is resonant if there exist $\alpha (1\le \alpha \le n-1)$ resonant relations such that

7 $${L}_{i}^{u}{\omega }_{i}=0\,\, u=1,...,\alpha$$

where ${L}_{i}^{u}$ are integers that are not all zero for a fixed *u*.

By using the canonical transformations of Eq. (5), the differential equations for the action and angle variables of the quasi-integrable Hamiltonian system (1) can be obtained from Eq. (1) as follows:

$$\dot{{I}_{r}}=\varepsilon \left(-{c}_{ij}\frac{\partial H}{\partial {P}_{j} }\frac{\partial {I}_{r}}{\partial {p}_{i}}+{h}_{ik}{\xi }_{k}\left(t\right)\frac{\partial {I}_{r}}{\partial {P}_{i}}\right)$$

8 $$\dot{{\theta }_{r}}={\omega }_{r}-\varepsilon {c}_{ij}\frac{\partial H}{\partial {P}_{j} }\frac{\partial {\theta }_{r}}{\partial {p}_{i}}+\varepsilon {h}_{ik}{\xi }_{k}(t)\frac{\partial {\theta }_{r}}{\partial {P}_{i}}$$

$$r,i,j=1,...,n;k=1,...,l$$

The form and dimension of the averaging equations depend on the resonance of the Hamiltonian system described in Eq. (8). In the following subsections, two cases are considered.

### External resonance only

Consider a system with external resonance but no internal resonance. Suppose that there are $\beta$ external primary resonant relations between the first $\beta$ oscillators and the first $\beta$ bounded excitations, i.e.,^27^

9 $${M}_{v}{\Omega }_{v}+{L}_{v}{\omega }_{v}=\varepsilon {\delta }_{v } \,\,\upsilon =1,...,\beta$$

where ${M}_{v}$ and ${L}_{v}$ are positive or negative integers and there is no summation over subscript $\upsilon .$ Introduce $\beta$ new variables:

10 $${\psi }_{v}={M}_{v}({\Omega }_{v}t+{\sigma }_{v}{B}_{v}(t)+{\Delta }_{v})+{L}_{v}{\theta }_{v}$$

Using the transformation in Eq. (10), the differential equations for ${I}_{1},...,{I}_{n, }{\psi }_{1}....,{\psi }_{n},{\theta }_{1}....,{\theta }_{n}$ can be obtained from Eq. (8) as follows:

$$\frac{d{I}_{r}}{dt}=\varepsilon \left[-{c}_{ij}\frac{\partial H}{\partial {p}_{j}}\frac{\partial {I}_{r}}{\partial {p}_{i}}+{h}_{ik1}\frac{\partial {I}_{r}}{\partial {p}_{i}}cos\left(\frac{1}{{M}_{k1}}{\psi }_{k1}-\frac{{L}_{k1}}{{M}_{k1}}{\theta }_{k1}\right)+{h}_{ik2}\frac{\partial {I}_{r}}{\partial {p}_{i}}{\xi }_{k2}(t)\right]$$

$$=\varepsilon {U}_{r}(I,\psi ,\theta )+\varepsilon {h}_{ik2}\frac{\partial {I}_{r}}{\partial {p}_{i}}{\xi }_{k2}(t)$$

$$\frac{d{\psi }_{v}}{dt}=\varepsilon \left[{\delta }_{v}-{L}_{v}{c}_{ij}\frac{\partial H}{\partial {p}_{j}}\frac{\partial {\theta }_{v}}{\partial {p}_{i}}+{L}_{v}{h}_{ik1}\frac{\partial {\theta }_{v}}{\partial {p}_{i}}cos(\frac{1}{{M}_{k1}}{\psi }_{k1}-\frac{{L}_{k1}}{{M}_{k1}}{\theta }_{k1})+{L}_{v}{h}_{ik2}\frac{\partial {\theta }_{r}}{\partial {p}_{i}}{\xi }_{k2}(t)\right]$$

$$+{M}_{v}{\sigma }_{v}\frac{d{B}_{V}(t)}{dt}$$

$$=\varepsilon {V}_{r}(I,\psi ,\theta )+\varepsilon {{L}_{v}h}_{ik2}\frac{\partial {\theta }_{v}}{\partial {p}_{i}}{\xi }_{k2}(t)+{M}_{v}{\sigma }_{v}\frac{d{B}_{v}(t)}{dt}$$

$$\frac{d{\theta }_{r}}{dt}={\omega }_{r}-\varepsilon {c}_{ij}\frac{\partial H}{\partial {p}_{j}}\frac{\partial {\theta }_{r}}{\partial {p}_{i}}+\varepsilon {h}_{ik1}{\xi }_{k}(t)\frac{\partial {\theta }_{r}}{\partial {p}_{i}})$$

11 $$r,i,j=1,...,n;{k}_{1},v=1,...,\beta ;{k}_{2}=\beta +1,...,l;k=1,...,l$$

where $$I=({I}_{1},...,{I}_{n}),\psi =({\psi }_{1},...,{\psi }_{\beta })$$,$$\theta =({\theta }_{1},...,{\theta }_{n})$$

As shown in Eq. (11), ${I}_{1},...,{I}_{n}$, and ${\psi }_{1},...,{\psi }_{\beta }$ are slowly-varying processes, while ${\theta }_{1},...,{\theta }_{n}$ are rapidly varying processes. By applying deterministic averaging to ${\theta }_{1},\dots ,{\theta }_{n}$, the averaged $IT\dot{O}$ equations can be defined as follows:

$$\mathrm{d}{I}_{r}=\varepsilon {\widetilde{U}}_{r}(I,\psi )dt \mathrm{d}{\psi }_{v}=\varepsilon {\widetilde{V}}_{r}(I,\psi )dt+{M}_{v}{\sigma }_{v}d{B}_{v}(t)$$

12 $$r=1,...,n;v=1,...,\beta$$

where

13 $${\widetilde{U}}_{r}=\frac{1}{(2\pi {)}^{n}}{\int }_{0}^{2\pi }{U}_{r}(I,\psi ,\theta )\,\,\,d\theta {\widetilde{V}}_{r}=\frac{1}{(2\pi {)}^{n}}{\int }_{0}^{2\pi }{V}_{r}(I,\psi ,\theta )d\theta$$

The averaged FPK equation associated with Eq. (12) is

14 $$\frac{\partial p}{\partial t}=-\varepsilon \frac{\partial ({\widetilde{U}}_{r}p)}{\partial {I}_{r}}-\varepsilon \frac{\partial ({\widetilde{V}}_{r}p)}{\partial {\psi}_{v}}+\frac{{M}_{v}^{2}{\sigma }_{v}^{2}}{2}\frac{{\partial }^{2}p}{\partial {\psi }_{v}^{2}}$$

where $p=p(I,\Psi ,0|{I}_{0},{\psi }_{0})$ is the transition probability density. The initial condition of Eq. (14) is

15 $$p=p(I,\Psi ,0|{I}_{0},{\psi }_{0})=\delta (I-{I}_{0})\delta |(\psi -{\psi }_{0})$$

The boundary conditions with respect to ${\psi }_{v}$ are periodic, i.e.,

16 $$p{|}_{{\Psi }_{n}+2n\pi }=p{|}_{{\psi }_{n}}) \,\,\,\,\,\frac{\partial p}{\partial {\psi }_{v}}{|}_{{\Psi }_{n}+2n\pi }=\frac{\partial p}{\partial {\psi }_{v}}{|}_{{\psi }_{n}}$$

$$u,v=1,...,\beta$$

The boundary conditions with respect to ${I}_{r}$ are defined as

17 $$p=finite \,\,at\,\, {I}_{r}=0$$

18 $$p,\frac{\partial p}{\partial {I}_{r}}\to 0 \,as \,\,{I}_{r}\to \infty$$

The reduced FPK equation with its boundary conditions can be solved numerically by using the combination of  finite difference method and the successive over-relaxation method.

### Both internal and external resonance

Consider a system with $\beta$ external resonant relations and $\alpha$ internal resonant relations, i.e.,

19 $${M}_{v}{\Omega }_{v}+{L}_{v}{\omega }_{v}=\varepsilon{\delta }_{v }\,\,\,\ \sum_{i=1}^{n}{N}_{i}^{u}{\omega }_{i}=\varepsilon {\sigma }_{u}\,\, \upsilon =1,...,\beta ;\,\,u=1,...,\alpha$$

where ${M}_{v}$ and ${L}_{v}$ are positive or negative integers and there is no summation over subscript $v$. The ${N}_{i}^{u}$ are also integers that are not all zero for a given $u$. Then new variables are introduced^37^:

$${\psi }_{v}={M}_{v}({\Omega }_{v}t+{\sigma }_{v}{B}_{v}(t)+{\Delta }_{v})+{L}_{v}{\theta }_{v}$$

20 $${\Phi }_{u}={\sum }_{i=1}^{n}{N}_{i}^{u}{\theta }_{i} \,\,\,\,v=1,\dots ,\alpha$$

The transformation is shown in Eq. (20), the differential equations for $I, \psi , \Phi ,$ and ${\theta }_{1}$ can be obtained from Eq. (8) as follows.

$$\frac{d{I}_{r}}{dt}=\varepsilon \left[-{c}_{ij}\frac{\partial H}{\partial {p}_{j}}\frac{\partial {I}_{r}}{\partial {p}_{i}}+{h}_{ik1}\frac{\partial {I}_{r}}{\partial {p}_{i}}cos(\frac{1}{{M}_{k1}}{\psi }_{k1}-\frac{{L}_{k1}}{{M}_{k1}}{\theta }_{k1})+{h}_{ik2}\frac{\partial {I}_{r}}{\partial {p}_{i}}{\xi }_{k2}(t)\right]$$

$$=\varepsilon {{U}^{^{\prime}}}_{r}(I,\psi ,\Phi ,{\theta }_{1})+\varepsilon {h}_{ik2}\frac{\partial {I}_{r}}{\partial {p}_{i}}{\xi }_{k2}(t)$$

$$\frac{d{\psi }_{v}}{dt}=\varepsilon \left[{\delta }_{v}-{L}_{v}{c}_{ij}\frac{\partial H}{\partial {p}_{j}}\frac{\partial {\theta }_{v}}{\partial {p}_{i}}+{L}_{v}{h}_{ik1}\frac{\partial {\theta }_{v}}{\partial {p}_{i}}cos(\frac{1}{{M}_{k1}}{\psi }_{k1}-\frac{{L}_{k1}}{{M}_{k1}}{\theta }_{k1})+{L}_{v}{h}_{ik2}\frac{\partial {\theta }_{r}}{\partial {p}_{i}}{\xi }_{k2}(t)\right]$$

$$+{M}_{v}{\sigma }_{v}\frac{d{B}_{V}(t)}{dt}$$

$$=\varepsilon {{V}^{^{\prime}}}_{r}(I,\psi ,\Phi ,{\theta }_{1})+\varepsilon {{L}_{v}h}_{ik2}\frac{\partial {\theta }_{v}}{\partial {p}_{i}}{\xi }_{k2}(t)+{M}_{v}{\sigma }_{v}\frac{d{B}_{v}(t)}{dt}$$

$$\frac{d{\Phi }_{u}}{dt}=\varepsilon \left({\sigma }_{u}-{N}_{{u}_{1}}^{u}{c}_{ij}\frac{\partial H}{\partial {p}_{j}}\frac{\partial {\theta }_{{u}_{1}}}{\partial {p}_{i}}+{N}_{{u}_{1}}^{u}{h}_{ik1}\frac{\partial {\theta }_{{u}_{1}}}{\partial {p}_{i}}\right)\times cos\left(\frac{1}{{M}_{k1}}{\psi }_{k1}-\frac{{L}_{k1}}{{M}_{k1}}{\theta }_{k1}\right)+{N}_{{u}_{1}}^{u}{h}_{ik2}\frac{\partial {\theta }_{{u}_{1}}}{\partial {p}_{i}}{\xi }_{{k}_{2}}=\varepsilon {{W}^{^{\prime}}}_{u}(I,\psi ,\Phi ,{\theta }_{1})+\varepsilon {{N}_{{u}_{1}}^{u}h}_{ik2}\frac{\partial {\theta }_{u1}}{\partial {p}_{i}}{\xi }_{k2}(t)$$

$$\frac{d{\theta }_{s}}{dt}={\omega }_{s}-\varepsilon {c}_{ij}\frac{\partial H}{\partial {p}_{j}}\frac{\partial {\theta }_{s}}{\partial {p}_{i}}+\varepsilon {h}_{ik}{\xi }_{k}(t)\frac{\partial {\theta }_{s}}{\partial {p}_{i}}$$

21 $$r,i,j,u=1,...,n;{k}_{1},v=1,...,\beta ;u=1,...,\alpha ;{k}_{2}=\beta +1,...,l;k=1,...,l; s=\alpha +1,...,n$$

where $I=({I}_{1},...,{I}_{n}),\psi =({\psi }_{1},...,{\psi }_{\beta })$, $\Phi =({\Psi }_{1},\dots ,{\Psi }_{\alpha }),\theta_{1} =({\theta }_{_{\alpha }},...,{\theta }_{n})$ and ${\theta }_{1} ,...,{\theta }_{n}$ are replaced by ${\Psi }_{1},...,{\Psi }_{\alpha }{,\theta }_{\alpha +1},...,{\theta }_{n}$.

In Eq. (21), $I$, $\psi$, and $\Phi$ are slowly varying processes, while ${\theta }_{1}$ is a rapidly varying process. By applying deterministic averaging to ${\theta }_{1}$ to Eq. (21), the averaging $IT\widehat{O}$ equations for $I$, $\psi$, and $\Phi$ can be expressed as:

$$\mathrm{d}{I}_{r}=\varepsilon {\widetilde{U}}_{r}(I,\psi ,\Phi )dt$$

$$\mathrm{d}{\psi }_{v}=\varepsilon {\widetilde{V}}_{r}(I,\psi ,\Phi )dt+{M}_{v}{\sigma }_{v}d{B}_{v}(t)$$

22 $$\mathrm{d}{\Phi }_{u}=\varepsilon {\widetilde{W}}_{u}(I,\psi ,\Phi )dt$$

$${\widetilde{U}}_{r}=\frac{1}{(2\pi {)}^{n-\alpha }}{\int }_{0}^{2\pi }{{U}^{^{\prime}}}_{r}(I,\psi ,\Phi ,\theta_{1} )d\theta_{1}\,\,\,\,\,\,\,\,\ {\widetilde{V}}_{v}=\frac{1}{(2\pi {)}^{n-\alpha }}{\int }_{0}^{2\pi }{{V}^{^{\prime}}}_{v}(I,\psi ,\Phi ,\theta_{1} )d\theta_{1}$$

23 $${\widetilde{W}}_{u}=\frac{1}{(2\pi {)}^{n-\alpha }}{\int }_{0}^{2\pi }{{W}^{^{\prime}}}_{u}(I,\psi ,\Phi ,\theta_{1} )d\theta_{1}$$

The averaging FPK equation associated with Eq. (22) is of the form

24 $$\frac{\partial p}{\partial t}=-\varepsilon \frac{\partial {\widetilde{U}}_{r}P}{\partial {I}_{r}}-\varepsilon \frac{\partial {\widetilde({V}}_{r}P)}{\partial {\psi }_{r}}-\varepsilon \frac{\partial {\widetilde{(W}}_{u}P)}{\partial {\Phi }_{u}}+\frac{{M}_{v}^{2}{\sigma }_{v}^{2}}{2}\frac{{\partial }_{p}^{2}}{\partial {\psi }_{v}^{2}}$$

Reduced averaged FPK equation (24) under similar boundary conditions can be solved numerically by using a finite difference method and the successive over-relaxation method.

## Motion model

To better introduce the case applications, we first provide a detailed description of the composition of the specific model and the parameter settings is provided.

### Theoretical hypothesis

In general, model complexity is determined according to the purpose of the model. The model should be sufficiently comprehensive to allow the reliable and accurate analysis of vibration response analyses in terms of ride comfort and safety. Motion stability of an EMS model refers to the parameters for a minimal coupling model composed of a maglev vehicle and a bridge including the elite segments proposed by Jin-hui Li ^6^. Depending on the basic elements analyzed, the complex systems is then simplified to minimum models, which is more efficient. Table 1 lists the correlation variables.

**Table 1:** Variables used in the model.

Variables			
*x*	Axial coordinate of the bridge	*N*	Number of coils
*T*	Time	*A*	Electromagnet area
*EI*_*B*_	Bending rigidity	*μ*_*0*_	Magnetic permeability of the vacuum
*ρ*_*B*_	Density of the bridge	*u*_*0*_	Initial voltage
*f(x,t)*	Electromagnetic forces, which depend on the vehicle location	*i*_*0*_	Initial current
*λ*_*B*_	Spatial wavelength of the first mode	*F*_*E0*_	Initial electromagnetic force
*F*_*Ei*_ (*t*)	Electromagnetic forces (*x* = *0.5L*_*B*_)	*k*_*p*_	Gap feedback coefficient
*Ω*	Spatial circular frequency of the guideway irregularity	*k*_*d*_	Gap first feedback derivative
*S*(*Ω*)	PSD (*mm*^*2*^*∙m*)	*k*_*ep*_	Equivalent magnetic dynamic stiffness
A ~ G	Spectral characteristic parameters	*k*_*ed*_	Equivalent magnetic dynamic damping
*α*^*2*^	Interference intensity of the Gaussian white noise	*ρ*	Canonical transformation variate
*β*	center frequency	*ξ*_*B*_	Damping ratio of the bridge
*σ*^*2*^	Variance of the guideway irregularity, with *ξ*(*x*) = *R*_*ξ*_(*0*)	*ω*_*B*_	Self-frequency of the bridge
*S*_*ξ*_(*ω*)	Spectral density of the shaping filter	R	Resistance
*m*_*E*_	Mass of the maglev vehicle	*−f*_*v*_	Aerodynamic drop
*m*_*B*_	Mass of the bridge	*ξ*_*1*_ (*t*)	Random irregularity
*y*_*E*_	Vertical displacement of the electromagnets	*σ*(*H*)	Diffusion coefficient
*y*_*B*_	Bridge vertical displacement	ρ	Canonical transformation variate
*L*_*E*_	Magnet length	$\delta$	Measurement gap between the electromagnet and bridge
+ *f*_*v*_	Aerodynamic lift	${\delta }_{0}$	Initial measurement gap
*B*(*t*)	Unit Wiener process	*H*(*t*)	Slowly varying stochastic process
*m*(*H*)	Drift coefficient	*α*_*1*_	Canonical transformation variate

The fundamental assumptions are described as follows:

- Electromagnet forces are linear.
- The system is decoupled both laterally and vertically without considering the turning radius, height difference or rolling freedom.
- The random irregularity is bounded noise applied with the shaping filter technique.
- A Bernoulli–Euler beam is adopted for the calculation of the bridge model.
- The moving mass and the action point of the concentrated force are at the geometric center of the electromagnets.

### Modelling of substructures

#### Bridge model

**Figure 1:**
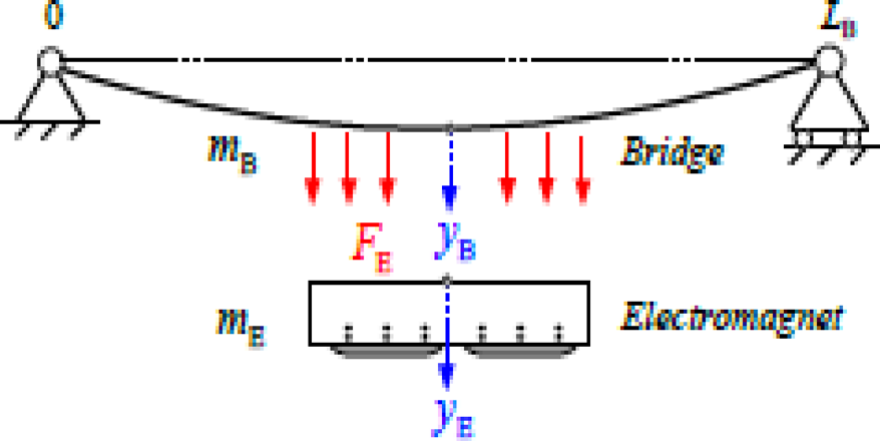
Minimal model.

Based on the above analysis, the minimal model is presented in Fig. 1^6, 10, 40^. The loads of vehicle and passengers are equivalent to a weight force acting on the center of the electromagnetic mass. The vehicle-bridge coupled system can be described using the structure is shown in Fig. 1. The electromagnetic forces are uniformly distributed on the bridge and the electromagnet. The current or voltage of the magnet that controls the electromagnetic action is applied to adjust the gap between the electromagnet and the bridge. The bridge is also shown in Fig. 1, where the endpoint marked with “0” is taken as the coordinate origin. The direction of the hammer is the positive direction of y-axis. Considering the high stiffness of the electromagnet, its deformation in the y-direction can be ignored. The dynamic characteristics have a considerable influence on the elastic deformation of the bridge in the y direction.

Based on the above assumptions, Fig. 1 illustrates a simplified suspension electromagnet-bridge coupling model. Where ${y}_{B}$ is the vertical displacement of the bridge, ${y}_{E}$ is the vertical displacement of the electromagnet relative to the reference plane, and $\delta$ is the distance between the electromagnet and the bridge.

The vertical motion of the bridge can be formulated as:

25 $$EI_{B} \frac{{\partial^{{4}} y_{B} (x,t)}}{{\partial x^{4} }} + \rho_{B} \frac{{\partial^{{2}} y_{B} (x,t)}}{{\partial t^{2} }} = f(x,t)$$

where $x$ is the transverse coordinate of the bridge, $E{I}_{B}$ is the bending stiffness of the bridge, ${\rho }_{B}$ is the linear density of the bridge, and $f(x,t)$ is the vertical force acting on the bridge.

$$\omega_{B} = \lambda_{B}^{{2}} \sqrt {EI_{B} {/}\rho_{{\mathrm{B}}} }$$

26 $$\phi_{B} {(}x{)} = sin{(}\lambda_{B} {(}x{)) }$$

When only the first mode is considered, ${\lambda }_{B}=\pi /{L}_{B}$. The solution to Eq. (1) can be expressed as,

27 $$y_{B} (x,t) = \phi _{B} (x)q_{B} (t)\ddot{q}_{B} (t) + 2\xi _{B} \omega _{B} \dot{q}_{B} (t) + \omega _{B}^{2} q_{B} (t) = 2\rho _{B}^{{ - 1}} L_{B}^{{ - 1}} \sum\limits_{{i = 1{\kern 1pt} :N_{E} }} {\phi _{B} (x_{i} )F_{{Ei}} (t)}$$

where ${q}_{\mathrm{B}}$ is the first-order generalized time domain coordinate of the bridge. By multiplying both sides of the above resultant equation by ϕB(x) *ϕ*_*B*(*x*)_ and then integrating both sides from 0 to *L*_*B*_ we obtain:

28 $$y_{B} (x,t) \, = \phi_{{\mathrm{B}}} (x)q_{B} (t)$$

The bridge’s vertical displacement equation as

29 $$\mathop {y_{B}}\limits^{..} (t) + 2\xi_{B} \omega_{B} \mathop {y_{B}}\limits^{.} (t) + \omega_{B}^{2} y_{B} (t) = 2m_{B}^{ - 1} \phi_{B}^{ - 1} \sum\limits_{{i = :N_{E} }} {\phi_{B} (x_{i} )F_{Ei} (t)}$$

#### Levitation model with feedback control

The electromagnetic forces can be simplified as long as the basic accuracy requirements are satisfied. Simplifications for the magnet-current relationship is linear near the ideal equilibrium point. The equivalent magnet dynamic stiffness and equivalent magnet dynamic damping are constant values [5]. The latter is related to the gap derivative. The equations of the electromagnetic forces as follows:

30 $$\left\{ \begin{gathered} F_{Ei } = F_{E0} + k_{ep} (\delta - \delta_{0} ) + k_{ed} \mathop \delta \limits^{.} \hfill \\ F_{E0} = \frac{{\mu_{0} N^{2} A}}{{4}}\left( {\frac{{i_{{0}} }}{{\delta_{{0}} }}} \right)^{{2}} \hfill \\ k_{ep} = \frac{{a_{1} }}{{\delta_{{0}}^{2} }} + \frac{{a_{2} }}{{\delta_{{0}}^{{3}} }} \hfill \\ k_{ed} = a_{3} k_{d} \frac{{u_{0} + k_{d} \mathop \delta \limits^{.} }}{{\delta_{{0}}^{2} }} \hfill \\ a_{1} = \frac{{\mu_{0} N^{2} Ak_{p} }}{2}(k_{p} \delta_{0} - i_{0} R) \hfill \\ a_{2} = - \frac{{\mu_{0} N^{2} A}}{2}(k_{p} \delta_{0} - i_{0} R)^{2} \hfill \\ a_{3} = \frac{{\mu_{0} N^{2} A}}{2} \hfill \\ \end{gathered} \right.$$

#### Guideway irregularity

The rail irregularity in a maglev line is the main source of extrinsic interference. At present, both the maglev lines in Shanghai^31^ and the Korean Institute of Machinery and Materials ^41^ implement their own measuring methods and have collected corresponding data. Due to the guideway irregularity caused by concrete shrinkage, concrete creep and vehicle loads, the wavelength is considered equal to the span of the bridge and the frequency is related to the vehicle speed.

Bounded noise includes harmonic variations with a maximum amplitude, a constant frequency, and random phases ^45–50^. It can be expressed via the stationary random process *ξ*_*1*_(*t*) as follows:

31 $$\xi_{1} (t) = A_{v} \cos (\Omega t + \sigma_{{1}} B(t) + \Delta )$$

where *Ω* is a constant center frequency with *Ω* = *π V/L,* with *V* being the vehicle speed and *L *being the bridge length,* A*_*v*_ is the maximum deflection of the bridge in the vertical direction, *t* is time, *σ*_*1*_ is the strength of the frequency perturbations, *B*(*t*) is a unit Wiener process, and *Δ* is a random phase that is uniformly distributed in [0, 2*π*]^37^. Its auto covariance functions are

32 $$\left\{ \begin{gathered} C_{{\xi_{1} (t)}} (\tau ) = \frac{{A_{v}^{{2}} \, }}{2}\exp ( - \frac{{\sigma^{2} |\tau {|}}}{{2}}){{\cos}}\Omega \tau \hfill \\ C_{{\mathop {\xi_{1} }\limits^{.} (t)}} (\tau ) = \frac{{{(}A_{v} \Omega )^{{2}} \, }}{2}\exp ( - \frac{{\sigma^{2} |\tau {|}}}{{2}}){{\cos}}\Omega \tau \hfill \\ \end{gathered} \right.$$

and their corresponding spectral densities are

33 $$\left\{ \begin{gathered} S_{\xi } \omega = \frac{{\sigma_{{1}}^{{2}} A_{v}^{{2}} }}{{{4}\pi }}[\frac{{\omega^{2} + \Omega^{2} + \sigma_{{1}}^{4} /4}}{{(\omega^{2} - \Omega^{2} - \sigma_{{1}}^{4} /4)^{{2}} + \sigma_{{1}}^{4} \omega^{2} }}] \hfill \\ S_{{\mathop \xi \limits^{.} }} \omega = \frac{{\sigma_{{1}}^{{2}} {(}A_{v} \Omega )^{{2}} }}{{{4}\pi }}[\frac{{\omega^{2} + \Omega^{2} + \sigma_{{1}}^{4} /4}}{{(\omega^{2} - \Omega^{2} - \sigma_{{1}}^{4} /4)^{{2}} + \sigma_{{1}}^{4} \omega^{2} }}] \hfill \\ \end{gathered} \right.$$

The variance of the bounded noise ^43^ is

34 $$\left\{ \begin{gathered} C_{\xi } ({0}) = \frac{{A_{v}^{2} }}{2} \hfill \\ C_{{\mathop \xi \limits^{.} }} ({0}) = \frac{{{(}A_{v} \Omega )^{2} }}{2} \hfill \\ \end{gathered} \right.$$

**Figure 2:**
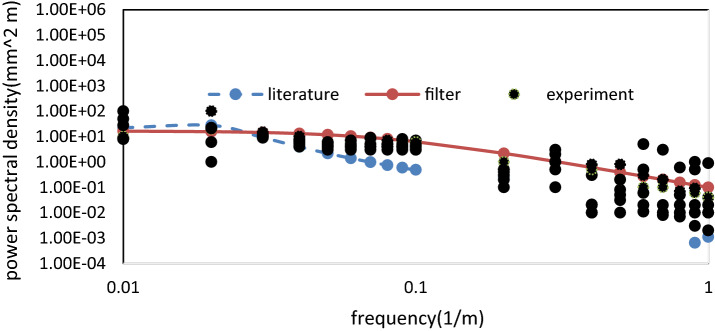
Comparison between the filter, the experiment line, and the literature reference.

A comparison between the filter, the experimental line, and the literature is shown in Fig. 2, where it is evident that there is numerical consistency.

### Dynamic differential equations

35 $$\left\{ \begin{gathered} m_{B} \mathop {y_{B} }\limits^{..} = { - }2m_{B} \xi_{B} \omega_{B} (\mathop {y_{B} }\limits^{.} + \mathop {\xi (t)}\limits^{.} ){ - }m_{B} \omega_{B}^{2} (y_{B} + \xi (t)) + \sigma [(F_{E0} + k_{ep} (y_{E} - y_{B} - \xi (t)) + k_{ed} (\mathop {y_{E} }\limits^{.} - \mathop {y_{B} }\limits^{.} - \mathop {\xi (t)}\limits^{.} )] \hfill \\ m_{E} \mathop {y_{E} }\limits^{..} = { - }\sigma [f_{mo} + k_{ep} (y_{E} - y_{B} - \xi (t)) + k_{ed} (\mathop {y_{E} }\limits^{.} - \mathop {y_{B} }\limits^{.} - \mathop {\xi (t)}\limits^{.} )] \hfill \\\sigma = {2}\phi_{{\mathrm{B}}} (x)\phi^{ - 1} (\pi x{/}L_{B} )\sum\limits_{i = 1}^{n} {\int_{{x_{{LE{\mathrm{i}}}} }}^{{x_{{LE{\mathrm{i}}}} + L_{E} }} {\phi_{{\mathrm{B}}}^{{2}} (x)dx} } \hfill \\ \end{gathered} \right.$$

In the above equations, $\sigma$ is the amplification factor of multiple suspension units. Through simplification, the dynamic differential equations can be expressed as:

36 $$\left\{ \begin{gathered} \hfill \\ \mathop {q_{1} }\limits^{.} = p_{1} \hfill\hfill \\ \mathop {p_{1} }\limits^{.} = \beta_{10} p_{1} + \beta_{11} (p_{2} - p_{1} ) + \alpha_{10} q_{1} + \alpha_{11} (q_{2} - q_{1} ) + \gamma_{10} + E_{11} \xi_{1} (t) + E_{12} \xi_{2} (t) \hfill \\ \mathop {q_{2} }\limits^{.} = p_{2} \hfill \\ \mathop {p_{2} }\limits^{.} = \beta_{20} p_{{2}} + \beta_{21} (p_{2} - p_{1} ) + \alpha_{20} q_{2} + \alpha_{21} (q_{2} - q_{1} ) + \gamma_{20} + E_{21} \xi_{{_{1} }} (t) + E_{22} \xi_{2} (t) \hfill \\ \end{gathered} \right.$$

where

$$\left\{ \begin{gathered} q_{1} = y_{B} ,q_{2} = y_{E}\, {, }\,p_{1} = {y_{B} }  \hfill \\ \beta_{20 } = 0\, {, }\, \beta_{{21 }}  \hfill \\ \xi_{1} (t) = \cos (\Omega t + \sigma_{{1}} B(t) + \Delta ),\xi_{2} (t) = sin(\Omega t + \sigma_{{2}} B(t) + \Delta ), \hfill \\ E_{11} = - \sigma k_{ep} A_{v} ,E_{12} = - \sigma k_{ed} A_{v} \Omega ,E_{21} = \sigma k_{ep} A_{v} ,E_{22} = \sigma k_{ed} A_{v} \Omega , \hfill \\ \end{gathered} \right.$$

## Analysis of the Lyapunov exponent and stationary probability

To better evaluate the case study, the calculation process is elaborated further.

The differential equations for the motion integrals *H*_*1*_ and *H*_*2*_ and the angle variables *θ*_*1*_ and *θ*_*2*_ are expressed as follows:

37 $$\begin{gathered} \left\{ \begin{gathered} \frac{{dH_{1} }}{dt} = p_{1} [(\beta_{10} - \beta_{11} )p_{1} + E_{11} \xi_{1} (t) + E_{12} \xi_{2} (t)] \hfill \\ \frac{{dH_{2} }}{dt} = p_{2} [(\beta_{20} + \beta_{21} )P_{2} + E_{21} \xi_{1} (t) + E_{22} \xi_{2} (t)] \hfill \\ \frac{{d\theta_{1} }}{dt} = \omega_{{1}} + \frac{{\partial \theta_{1} }}{{\partial p_{1} }}[(\beta_{10} - \beta_{11} )P_{1} + E_{11} \xi_{1} (t) + E_{12} \xi_{2} (t)] \hfill \\ \frac{{d\theta_{2} }}{dt} = \omega_{2} + \frac{{\partial \theta_{2} }}{{\partial p_{2} }}[(\beta_{20} + \beta_{21} )P_{2} + E_{21} \xi_{1} (t) + E_{22} \xi_{2} (t)] \hfill \\ \end{gathered} \right. \hfill \\ \hfill \\ \end{gathered}$$

where

$$\left\{ \begin{gathered} H_{1 } = \frac{{p_{{1}}^{2} }}{2} - \omega_{B}^{2} q_{1}^{{_{2} }} {/2} - \alpha_{11} (q_{{1}} { - }q_{{2}} )^{{2}} {/2} + \gamma_{{{10}}} q_{{1}} \hfill \\ H_{2 } = \frac{{p_{{2}}^{2} }}{2} - \omega_{E}^{2} q_{2}^{{_{2} }} {/2} + \alpha_{21} (q_{{1}} { - }q_{{2}} )^{{2}} {/2} + \gamma_{{{20}}} q_{{2}} \hfill \\ \end{gathered} \right.$$

### Only external resonant vibration

Consider a system with external resonance but no internal resonance. Suppose that there is a single external resonant relation ^36^.

38 $$\Omega_{1} { - 2}\omega_{{1}} = \varepsilon \Theta$$

where *ε* and *Θ* can be regarded as small detuning parameters. The new variable *ψ* is introduced, as defined in Eq. (39).

39 $$\psi = \Omega_{{1}} t + \sigma_{1} B_{1} (t) + \Delta_{1} - 2\theta_{1}$$

The differential equations for *H*_*1*_, *H*_*2*_, and *ψ*, are stated below:

40 $$\left\{ \begin{gathered} dH_{1} = [(\beta_{10} - \beta_{11} )H_{1} + \frac{{E_{11} }}{{2\omega_{1} }}sin\psi H_{1} + \frac{{E_{12} }}{{2\omega_{1} }}cos\psi H_{1} )]dt \hfill \\ dH_{2} = [(\beta_{20} + \beta_{21} )H_{{2}} + \frac{{E_{21} }}{{2\omega_{2} }}sin\psi H_{2} + \frac{{E_{22} }}{{2\omega_{2} }}cos\psi H_{2} ]dt \hfill \\ d\psi = [(\Omega_{1} - 2\omega_{1} ) + \frac{{E_{11} }}{{2\omega_{1} }}\cos \psi + \frac{{E_{12} }}{{2\omega_{1} }}sin\psi ]dt + \sigma_{1} dB_{1} (t) \hfill \\ \end{gathered} \right.$$

The differential equations for $\rho$ and $\alpha_{{1}}$ can be formulated as follows

41 $$\left\{ \begin{gathered} d\rho = \frac{{1}}{{2}}(\beta_{{{20}}} + \beta_{{{21}}} + ( - \beta_{{2{0}}} - \beta_{{{21}}} - \frac{{E_{21} }}{{2\omega_{2} }}\sin \psi - \frac{{E_{22} }}{{2\omega_{2} }}\cos \psi + \beta_{{{10}}} - \beta_{{{11}}} + (\frac{{E_{11} }}{{2\omega_{1} }}\sin \psi + \frac{{E_{21} }}{{2\omega_{1} }}\cos \psi ))\alpha_{1} \hfill \\ \begin{array}{*{20}c} {} & {} \\ \end{array} + \sqrt {\alpha_{1} (1 - \alpha_{1} )} + (\frac{{\alpha_{11} }}{{\omega_{2} }} - \frac{{\alpha_{21} }}{{\omega_{1} }}{)}sin\varphi )dt \hfill \\ d\alpha_{1} = ( - \beta_{{2{0}}} - \beta_{{{21}}} - \frac{{E_{21} }}{{2\omega_{2} }}\sin \psi - \frac{{E_{22} }}{{2\omega_{2} }}\cos \psi + \beta_{{{10}}} - \beta_{{{11}}} + (\frac{{E_{11} }}{{2\omega_{1} }}\sin \psi + \frac{{E_{21} }}{{2\omega_{1} }}\cos \psi ))\alpha_{1} (1 - \alpha_{1} )dt \hfill \\ \end{gathered} \right.$$

The averaging FPK equation presented by Zhu (2002) [17] that is associated with $\psi$ is

42 $$\sigma_{{1}}^{{2}} \frac{{d^{2} p}}{{d\psi^{2} }} - 2\frac{d}{d\psi }[((\Omega_{1} - {2}\omega_{{1}} ) + (\frac{{E_{{{11}}} }}{{2\omega_{{1}} }}cos(\psi ) + \frac{{E_{{{12}}} }}{{2\omega_{{1}} }}sin(\psi ))p] = 0$$

The solution that satisfies the periodic condition is

43 $$p(\psi ) = C\exp \{ \frac{2}{{\sigma_{1}^{2} }}[(\Omega_{1} - \omega_{{1}} )\psi + \frac{{E_{11} }}{{2\omega_{1} }}sin(\psi ) + \frac{{E_{12} }}{{2\omega_{1} }}cos(\psi )]\} \int_{\psi }^{2\pi + \psi } {\exp \{ - \frac{2}{{\sigma_{1}^{2} }} \times [(\Omega_{1} - } \omega_{{1}} )\psi + \frac{{E_{11} }}{{2\omega_{1} }}sin(\psi ) + \frac{{E_{12} }}{{2\omega_{1} }}cos(\psi )]d\psi$$

where C is a normalized constant. The mean $d\alpha_{1}$ can be calculated as:

44 $$d\alpha_{1} = ( - \beta_{{2{0}}} - \beta_{{2{1}}} - \frac{{E_{21} }}{{2\omega_{2} }}\sin \psi - \frac{{E_{22} }}{{2\omega_{2} }}\cos \psi + \beta_{{{10}}} - \beta_{{{11}}} + (\frac{{E_{11} }}{{2\omega_{1} }}\sin \psi + \frac{{E_{21} }}{{2\omega_{1} }}\cos \psi ))\alpha_{1} (1 - \alpha_{1} )dt = A(\alpha )dt$$

If $A(\alpha ) > 0$, $\alpha_{{1}} \to {1}$ and $p(\alpha_{1} ,\psi ) = p(\psi )\delta (1)$. if $A(\alpha ) < 0$, $\alpha_{{1}} \to {0}$ and $p(\alpha_{1} ,\psi ) = p(\psi )\delta ({0})$^.^

The equation can be expressed as:

45 $$\lambda_{{1}} = \int_{{0}}^{{{2}\pi }} {\int_{{0}}^{{1}} {\frac{{1}}{{2}}} } A{(}\alpha {)}p{(}\alpha_{{1}} ,\psi )d\alpha_{1} d\psi$$

**Figure 3:**
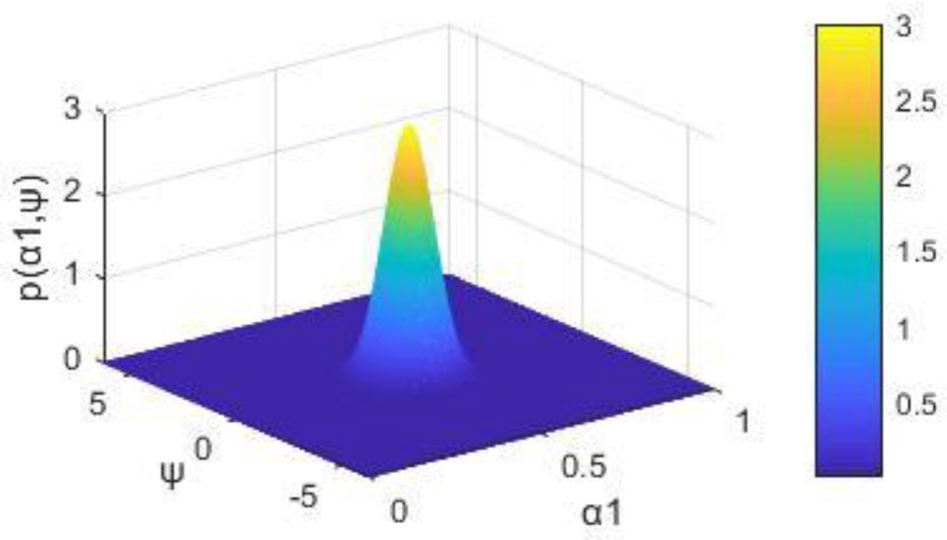
Stationary probability densities in a system with external excitation only.

**Figure 4:**
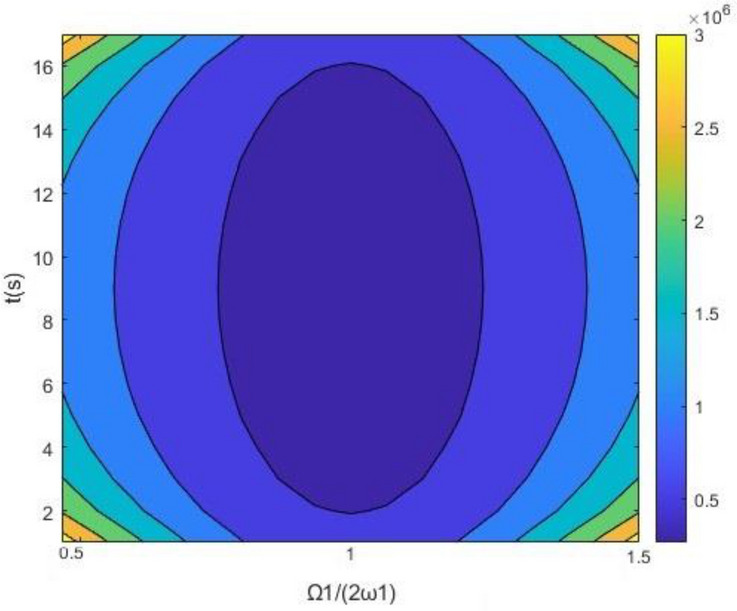
Time–frequency-amplitude region of cross stability in a system with external excitation only.

**Figure 5:**
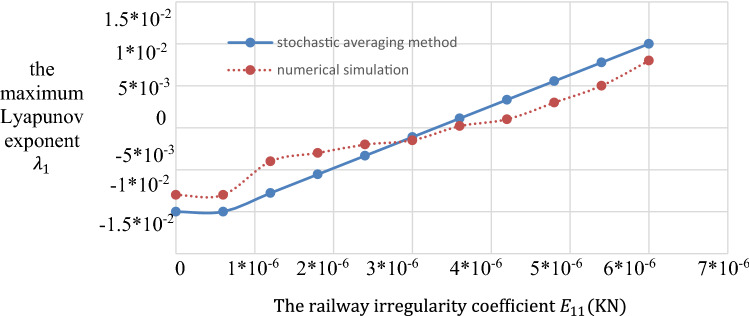
Lyapunov exponent in a system with external excitation only.

Some results we obtained via simulations. The joint probability density *p*(*α*_*1*,_ *ψ*) represents the centralized peak when *Ψ* = 0 and *α*_*1*_ = 0. In Fig. 3, when *ψ* = 0 and $\alpha_{{1}} = 0.5$, the stationary joint probability density *p*(*α*_*1*,_ *ψ*) shows a peak. In Fig. 3, when $\Omega_{1} { - 2}\omega_{{1}} = 0$, the first time of resonating to exceed is the shortest. As shown in Fig. 3 and Fig. 4, the cross-stable region in the frequency-excitation amplitude plane has a valley shape when $\lambda_{{1}} = {0}$. As the guideway irregularity coefficient E_11_ increases, the maximum Lyapunov exponents increase gradually from their initial small stable state, as shown in Fig. 5. A comparison between the stochastic averaging method and the numerical simulation is also shown in Fig. 5, where the numerical consistency between the results is evident.The random average method is more vivid from the grasp of the critical value of total energy and the changing trend. Through the grasp of displacement, the numerical simulation has a large amount of calculation.

### Both internal and extrinsic resonance

Consider a case with primary external resonance between the first bounded noise excitation and the first oscillator. The primary internal resonance between the two oscillators [36] can be expressed as:

46 $$\left\{ \begin{gathered} \Omega_{{1}} { - 2}\omega_{{1}} = \varepsilon \Theta \hfill \\ \omega_{{2}} { - }\omega_{{1}} = \varepsilon \eta \hfill \\ \end{gathered} \right.$$

where $\Theta$ and $\eta$ are detuning parameters ^26^. The new variables $\psi$ and $\Phi$ are introduced as angle differences.

47 $$\left\{ \begin{gathered} {{\psi = \Omega }}_{{1}} {{t + \sigma }}_{{1}} {{\rm B}}_{{1}} {{(t) + \Delta }}_{{1}} {{ - 2\theta }}_{{1}} \hfill \\ \Phi {{ = \theta }}_{{2}} {{ - \theta }}_{{1}} \, \hfill \\ \end{gathered} \right. \,$$

The differential equations for $H_{1}$, $H_{2}$, $\psi$, and $\Phi$ can be formulated as

48 $$\left\{ \begin{gathered} dH_{1} = [(\beta_{10} - \beta_{11} )H_{1} + \frac{{E_{11} }}{{2\omega_{1} }}sin\psi H_{1} + \frac{{E_{12} }}{{2\omega_{1} }}cos\psi H_{1} + \frac{{\alpha_{11} \sqrt {H_{1} H_{2} } }}{{\omega_{2} }}sin\phi )]dt \hfill \\ dH_{2} = [(\beta_{20} + \beta_{21} )H_{{2}} + \frac{{E_{21} }}{{2\omega_{2} }}sin\psi H_{2} + \frac{{E_{22} }}{{2\omega_{2} }}cos\psi H_{2} - \frac{{\alpha_{21} \sqrt {H_{1} H_{2} } }}{{\omega_{1} }}sin\phi ]dt \hfill \\ d\psi = [(\Omega_{1} - 2\omega_{1} ) + \frac{{E_{11} }}{{2\omega_{1} }}\cos \psi + \frac{{E_{12} }}{{2\omega_{1} }}sin\psi + \frac{{\alpha_{11} }}{{\omega_{2} }}\sqrt {\frac{{H_{2} }}{{H_{1} }}} \cos \phi ]dt + \sigma_{1} dB_{1} (t) \hfill \\ d\phi = [(\omega_{2} - \omega_{1} ) + \frac{{E_{11} }}{{4\omega_{1} }}\cos \psi + \frac{{E_{12} }}{{4\omega_{1} }}sin\psi + \frac{{\alpha_{11} }}{{\omega_{2} }}\sqrt {\frac{{H_{2} }}{{H_{1} }}} \cos \phi - \frac{{\alpha_{21} }}{{\omega_{1} }}\sqrt {\frac{{H_{1} }}{{H_{2} }}} \cos \phi ]dt \hfill \\ \end{gathered} \right.$$

The differential equations for $\rho$ and $\alpha_{{1}}$ can be formulated as

49 $$\left\{ \begin{gathered} d\rho = \frac{{1}}{{2}}(\beta_{{{11}}} + \beta_{{{21}}} +( \beta_{{2{0}}} + \beta_{{{10}}} { - }\beta_{{{11}}} { - }\beta_{{{21}}} + \frac{{E_{11} }}{{2\omega_{1} }}\sin \psi + \frac{{E_{12} }}{{2\omega_{1} }}\cos \psi - \frac{{E_{21} }}{{2\omega_{2} }}\sin \psi - \frac{{E_{22} }}{{2\omega_{2} }}\cos \psi )\alpha_{1} \hfill \\ \begin{array}{*{20}c} {} & {} \\ \end{array} + (\frac{{\alpha_{11} }}{{\omega_{2} }} - \frac{{\alpha_{21} }}{{\omega_{2} }})\sin \Phi \sqrt {\alpha_{1} (1 - \alpha_{1} )} )dt \hfill \\ d\alpha_{1} = \beta_{{2{0}}} + \beta_{{{10}}} { - }\beta_{{{11}}} { - }\beta_{{{21}}} + \frac{{E_{11} }}{{2\omega_{1} }}\sin \psi + \frac{{E_{12} }}{{2\omega_{1} }}\cos \psi - \frac{{E_{21} }}{{2\omega_{2} }}\sin \psi - \frac{{E_{22} }}{{2\omega_{2} }}\cos \psi )\alpha_{1} (1 - \alpha_{1} ) + (\sqrt {\alpha_{1} (1 - \alpha_{1} )} \hfill \\ \begin{array}{*{20}c} {} & {} \\ \end{array} (1 - \alpha_{1} )\frac{{\alpha_{11} }}{{\omega_{2} }}\sin \Phi - \sqrt {\alpha_{1} (1 - \alpha_{1} )} \alpha_{1} \frac{{\alpha_{21} }}{{\omega_{2} }}\sin \Phi )dt \hfill \\ \end{gathered} \right.$$

*p*(*α*_*1*_*, ψ, Ф*) can be derived from the following equation, the derivation of which can be found in ^17^.

50 $$\frac{\partial p}{{\partial t}} = \frac{{\partial m_{1} }}{{\partial \alpha_{1} }} + \frac{\partial G}{{\partial \psi }} + \frac{\partial K}{{\partial \Phi }} + \frac{{\sigma_{1}^{2} }}{2}\frac{{\partial p^{2} }}{{\partial \psi^{2} }}$$

The transition probability density is obtained from the solution of the FPK equation:$p = p(\alpha_{1} ,\psi ,\Phi ,t|\alpha_{0} ,\psi_{0} ,\Phi_{0} )$.

The maximum Lyapunov exponent can be expressed as:

51 $$\begin{gathered} \lambda_{{1}} = \int_{{0}}^{{1}} {\int_{{0}}^{{{2}\pi }} {\int_{{0}}^{{{2}\pi }} {\frac{{1}}{{2}}} } } (\beta_{{{11}}} + \beta_{{{21}}} +( \beta_{{2{0}}} + \beta_{{{10}}} { - }\beta_{{{11}}} { - }\beta_{{{21}}} + \frac{{E_{11} }}{{2\omega_{1} }}\sin \psi + \frac{{E_{12} }}{{2\omega_{1} }}\cos \psi - \frac{{E_{21} }}{{2\omega_{2} }}\sin \psi - \frac{{E_{22} }}{{2\omega_{2} }}\cos \psi )\alpha_{1} \hfill \\ \begin{array}{*{20}c} {} & {} \\ \end{array} + (\frac{{\alpha_{11} }}{{\omega_{2} }} - \frac{{\alpha_{21} }}{{\omega_{2} }})\sin \Phi \sqrt {\alpha_{1} (1 - \alpha_{1} )} )]p(\alpha_{1} ,\psi ,\phi )d\alpha_{1} d\psi d\phi \hfill \\ \end{gathered}$$

**Figure 6:**
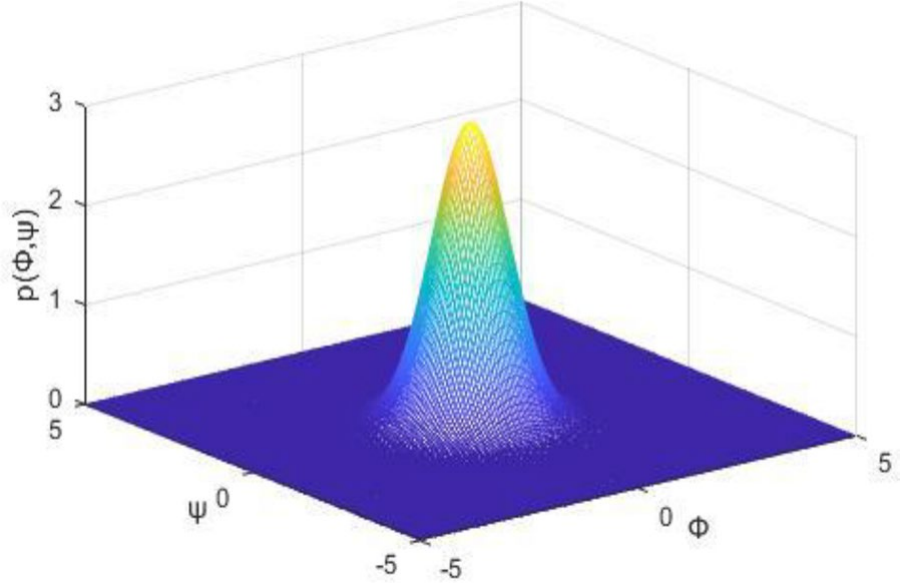
Stationary probability density of a system with both internal and external excitation.

**Figure 7:**
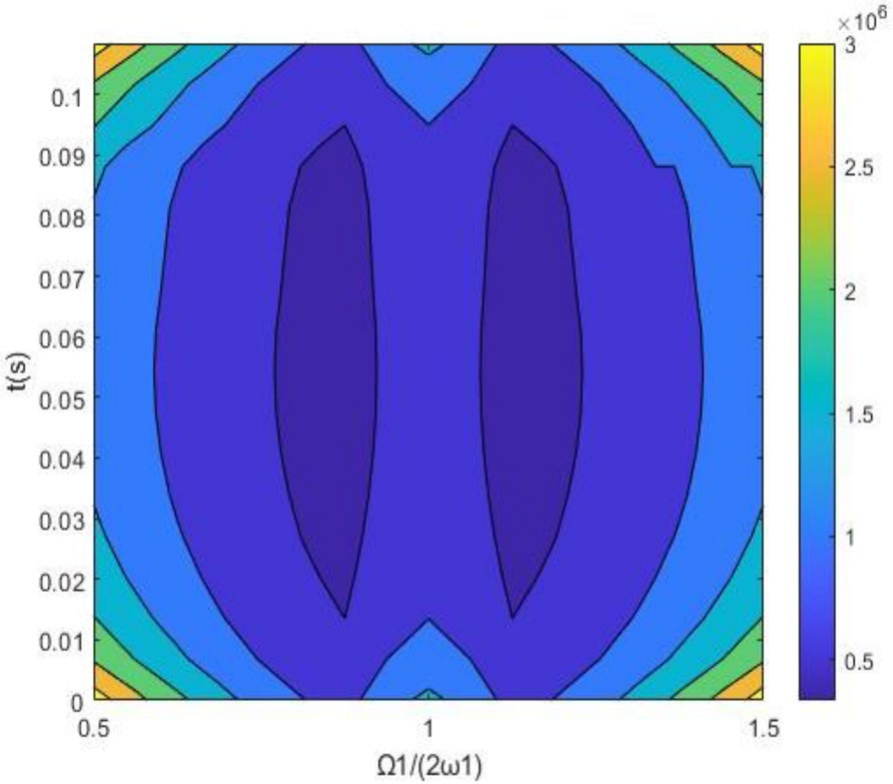
Time–frequency amplitude stable region in a system with both internal and external excitation.

**Figure 8:**
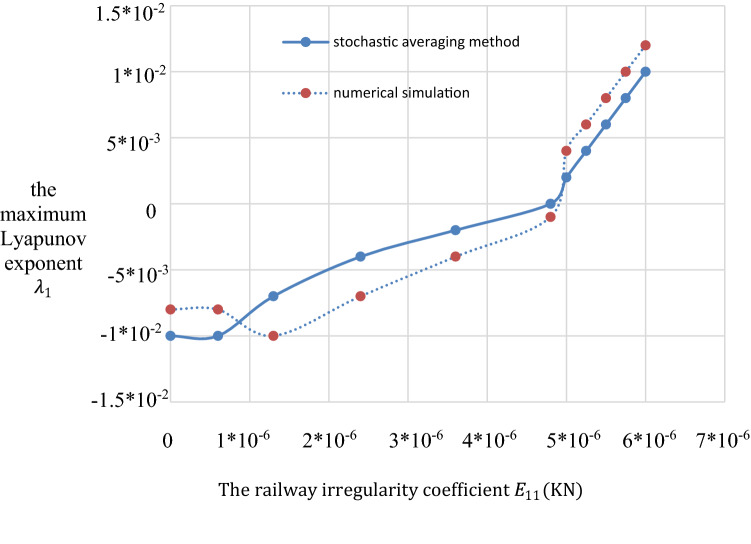
Lyapunov exponents of a system with both internal and external excitation.

A numerical calculation is helpful for determining reason for this resonance. In Fig. 6, when *ψ* = 0, $\Phi$ = 0, the stationary joint probability density *p*(*Φ*_,_ *ψ*) shows a peak. The joint probability density *p*(*Φ*_,_ *ψ*) represents a centralized distribution with the angle differences *Ψ* = 0 and *Φ* = 0. Figures 6 and 7 show the stable and unstable regions in the frequency-excitation amplitude plane, which is resembles a saddle shape. As the guideway irregularity coefficient E_11_ increases, the maximum Lyapunov exponents start from their initial small stable state and rise in a step-wise manner, as shown in Fig. 8. A comparison between the stochastic averaging method and the numerical simulation is also shown in Fig. 8, where numerical consistency between the stochastic averaging method and the numerical simulation is also shown in Fig. 8, where numerical consistency can be observed. The random average method is more vivid from the grasp of the critical value of total energy and the changing trend. Through the grasp of displacement, the numerical simulation has a large amount of calculation.

## Summary

In this paper, stochastic averaging method for a quasi-integrable Hamiltonian system under bounded noise is proposed in this paper. The forms and dimensions of the averaging equations depend on the number of internal and external resonant relations in the system. The proposed procedures were applied in the prediction of a high-speed maglev train-bridge coupled system responses under bounded noise. The results obtained from the reduced averaging FPK equation by using the finite difference and the successive over-relaxation iterative methods are consistent with simulations of the original system. It is noted that the proposed procedure may also be applicable in studying the reliability and stochastic stability of these systems under bounded noise. The results conclusively show that

- The joint probability density of different phases has a peak when the phases are close to each other.
- The stable region shrinks when the two resonance conditions are satisfied.
- When the unstable region in the phase diagram (E_11_, Ω_/_2ω_1_) is affected by only one external resonance, the external resonance reduces the stable region. The closer the external resonance frequency is to the system frequency, the smaller the size of the stable region. Moreover, as E_11_ increases, the maximum Lyapunov exponent changes from negative to positive, and the system shifts from stability to instability in a nearly linear manner.
- When the unstable region in the phase diagram (E_11_, Ω_/_2ω_1_) is affected by both internal and external resonance, the stable region shrinks as the energy is transferred from the first oscillator to the second oscillator during the two resonances. As E_11_ increases, the maximum Lyapunov exponent changes from negative to positive, and the system shifts from stability to instability in a step-wise manner.

## Data Availability

The data used in this study can be obtained from the corresponding author upon request.
